# Bioactive Potential of Extracts of *Labrenzia aggregata* Strain USBA 371, a Halophilic Bacterium Isolated from a Terrestrial Source

**DOI:** 10.3390/molecules25112546

**Published:** 2020-05-29

**Authors:** Carolina Díaz-Cárdenas, Laura Yinneth Rojas, Susana Fiorentino, Monica P. Cala, Jorge I Díaz, Freddy A. Ramos, Jean Armengaud, Silvia Restrepo, Sandra Baena

**Affiliations:** 1 Unidad de Saneamiento y Biotecnología Ambiental, Departamento de Biología, Pontificia Universidad Javeriana, P.O. Box 56710 Bogotá DC, Colombia; diazcardenascaro@gmail.com; 2 Grupo de Inmunobiología y Biología Celular, Pontificia Universidad Javeriana, P.O. Box 56710 Bogotá DC, Colombia; rojasl.a@javeriana.edu.co (L.Y.R.); susana.fiorentino@javeriana.edu.co (S.F.); 3 Metabolomics Core Facility—MetCore, Vicepresidency for Research and Creation, Universidad de los Andes, Cra 1 No. 18A-12, 111711 Bogotá DC, Colombia; mp.cala10@uniandes.edu.co; 4 Vicepresidency of Research and Creation, Universidad de los Andes, Cra 1 No. 18A-12, 111711 Bogotá DC, Colombia; ji.diaz1@uniandes.edu.co; 5 Departamento de Química, Facultad de Ciencias, Universidad Nacional de Colombia-Sede Bogotá, Carrera 30 # 45-03, 110111 Bogotá DC, Colombia; faramosr@unal.edu.co; 6 Département Médicaments et Technologies pour la Santé (DMTS), CEA, INRAE, Université Paris Saclay, SPI, 30200 Bagnols-sur-Cèze, France; jean.armengaud@cea.fr; 7 Chemical Engineering Department, Universidad de los Andes, Cra 1 No. 18A-12, 111711 Bogotá DC, Colombia; srestrep@uniandes.edu.co

**Keywords:** *Labrenzia aggregata* USBA 371, cytotoxic activities, bioactive natural products, saline natural products, secondary metabolism, genome annotation, NMR characterization

## Abstract

Previous studies revealed the potential of *Labrenzia aggregata* USBA 371 to produce cytotoxic metabolites. This study explores its metabolic diversity and compounds involved in its cytotoxic activity. Extracts from the extracellular fraction of strain USBA 371 showed high levels of cytotoxic activity associated with the production of diketopiperazines (DKPs). We purified two compounds and a mixture of two other compounds from this fraction. Their structures were characterized by 1D and 2D nuclear magnetic resonance (NMR). The purified compounds were evaluated for additional cytotoxic activities. Compound **1** (cyclo (l-Pro-l-Tyr)) showed cytotoxicity to the following cancer cell lines: breast cancer 4T1 (IC_50_ 57.09 ± 2.11 µM), 4T1H17 (IC_50_ 40.38 ± 1.94), MCF-7 (IC_50_ 87.74 ± 2.32 µM), murine melanoma B16 (IC_50_ 80.87 ± 3.67), human uterus sarcoma MES-SA/Dx5 P-pg (−) (IC_50_ 291.32 ± 5.64) and MES-SA/Dx5 P-pg (+) (IC_50_ 225.28 ± 1.23), and murine colon MCA 38 (IC_50_ 29.85 ± 1.55). In order to elucidate the biosynthetic route of the production of DKPs and other secondary metabolites, we sequenced the genome of *L. aggregata* USBA 371. We found no evidence for biosynthetic pathways associated with cyclodipeptide synthases (CDPSs) or non-ribosomal peptides (NRPS), but based on proteogenomic analysis we suggest that they are produced by proteolytic enzymes. This is the first report in which the cytotoxic effect of cyclo (l-Pro-l-Tyr) produced by an organism of the genus *Labrenzia* has been evaluated against several cancer cell lines.

## 1. Introduction

More than one third of the approved drugs correspond to natural products or have been derived from compounds found in living organisms [1]. The discovery of new compounds from halophilic and halotolerant microorganisms has become increasingly important in natural products research [2,3,4,5,6] as these microorganisms represent reservoirs of novel bioactive metabolites with diverse groups of chemical structures [7]. Compounds isolated from deep-sea microorganisms could represent new anti-tumor drugs of interest [6], but organisms from similar environments such as saline ecosystems could also be considered as anti-tumor drug natural repositories, as shown in recent studies [2]. 

The *Rhodobacteraceae* family has been isolated mainly from marine habitats, in association with marine invertebrates such as corals, colonizing the surfaces of oysters and shells and in the rhizosphere of halophytes plants from coastal areas [8,9,10]. *Labrenzia* spp., a member of this family, possess biosynthetic genes for the production of non-ribosomal peptides (NRPS), polyketide synthases (PKS), bacteriocins and terpenoids [9,11], but few secondary metabolites have been isolated. To date, the described compounds are a polyketide pederin analogue, isolated from a free-living marine *Labrenzia* sp. PHM005, with cytotoxic activity against cancer cell lines A549 (ATCC CCL-185) (lung carcinoma, NSCLC); HT-29 (ATCC HTB-38) (colon adenocarcinoma); MDA-MB-231 (ATCC HTB-26) (breast adenocarcinoma) and PSN-1 (ATCC CRL-3211) (pancreas adenocarcinoma) [12], and a new catecholate-class siderophore designated Labrenzbactin with antimicrobial activity against *Micrococcus luteus* ATCC9341 and *Ralstonia solanacearum* SUPP1541 and cytotoxicity against P388 murine leukemia cells [13]. Cyclopropane fatty acids, derived from the primary metabolism of other marine *Labrenzia* spp., with activity against resistant multidrug pathogenic bacteria and fungi have been detected [14]. However, our knowledge on compounds produced by members of the genus *Labrenzia* is still limited, and there is a need to explore the metabolic potential of this halophilic genus as source of natural molecules with biological activity.

In a previous study [15], several cytotoxic compounds from more than 50 halophilic and halotolerant bacterial strains isolated from different Colombian saline environments were characterized. We described that *Labrenzia aggregata* USBA 371 cultured in saline tryptic soy broth (TSB) media supplemented with 3% (*w*/*v*) NaCl produced a mixture of compounds, predominantly DKPs that were secreted to the extracellular medium and had cytotoxic activity. The cytotoxic effect of USBA 371 was related to the action of a diverse mixture of DKPs namely cyclo (Pro-Phe), cyclo (Leu-Phe), cyclo (Val-Phe), cyclo (Phe-Phe), cyclo (Pro-Leu), cyclo (His-Pro), cyclo (Pro-Val), cyclo (Pro-Ala) and cyclo (Pro-Tyr). The extract containing these DKPs presented a high cytotoxic effect against the adherent murine mammary cell carcinoma 4T1 and human mammary adenocarcinoma MCF-7 cell lines (IC_50_ < 5.5 μg/mL) [15]. In addition to DKPs, this mix contained other compounds that did not match any of the databases used [15], indicating the need to further explore the metabolic diversity of the strain and the cytotoxic activity of these new compounds.

In the present study, we performed a metabolic profiling study of crude extracts from *L. aggregata* USBA 371 with high cytotoxic activity followed by the isolation and identification of the compounds responsible for the biological activity previously detected. To shed light on the metabolic potential of this strain, we sequenced and annotated its genome to identify its biosynthetic gene clusters. Using a proteogenomics approach, we detected the expression of proteins associated to enzyme complexes involved in the production of secondary metabolites. We show that *L. aggregata* USBA 371 is an important source of DKPs and characterize their cytotoxic activity, which had not been previously described in this halophilic genus.

## 2. Results and Discussion 

### 2.1. Metabolic Profiling of Crude Extracts from Chloroform Extractions 

As our earlier study [15] showed that a chloroform extract from the cell-free supernatants of *L. aggregata* USBA 371 culture was highly cytotoxic against 4T1 and MCF7 cell lines, we decided to identify the principal metabolites of crude extracts obtained by sequential extraction with chloroform and ethyl acetate. 

The LC/MS analysis of crude extracts obtained from chloroform extraction detected the presence of 29 putative compounds associated to peptides, alkaloids, fatty acids, glycerolipids and glycerophospholipids, as shown in Table 1. 

The crude extract compounds were mainly the DKPs cyclo (l-Leu-l-Phe), cyclo (l-Leu-l-Leu), cyclo (l-Pro-l-Tyr) and cyclo (l-Ala-l-Pro). These are cyclic dipeptides produced as secondary metabolites by bacteria, fungi, plants and mammals [16,17]. Natural products containing DKP scaffolds are structurally diverse and resistant to proteolysis [18], and are known to have different functions. They are involved in quorum sensing [19] and have antifungal [20], antibacterial [20,21], anti-inflammatory [22] and cytotoxic activities [23,24], and are gaining great interest due to their bioactive potential [25]. This metabolic profiling confirms our previous results where we found high amounts of DKP in extracts from strain USBA 371 cultured in TSB 3% (*w*/*v*) NaCl [15]. 

We isolated two DKPs and identified them as cyclo (l-Pro-l-Tyr) and cyclo (l-Tyr-l-Phe). They are hereafter referred to as compound **1** (Figures S1–S5) and compound **2** (Figures S6 and S7), respectively. We also isolated a fraction containing a mix of cyclo (l-Phe-l-Pro) (compound **3a**) and cyclo (l-Ala-l-Pro) (compound **3b**) (Figures S8 and S9). The compounds were identified by NMR spectroscopy, and their data were compared to published data [20,22,25,26,27]. Compound **1** corresponds to maculosin-1 which has been reported as a secondary metabolite of bacteria and fungi [20,28]. 

### 2.2. Cytotoxic Activity Analysis of Major Compounds in Chloroform Extracts

The cytotoxic activity of the crude extract, compounds **1** and **2**, and the mix of compounds **3a** and **3b** was assessed against cell lines 4T1 and MCF-7. The cytotoxic analysis showed activity in the **3a** and **3b** compound mix (IC_50_ < 16.1 ± 1.6 µg/mL) and in compound **1** (IC_50_ < 88 µM), while compound **2** showed reduced cytotoxic activity (IC_50_ > 250 μM) (Table 2 and Figure S10A,B). 

Our results show that the crude extract and a mixture of compounds **3a** and **3b** have greater cytotoxic effects than do the pure compounds. This could be due to the synergistic or additive activity which has been widely documented in plant extracts [29,30]. The search for compounds with antimalarial or antitumor activity has found that biological activity can be lost in extracts through processes to isolate active compounds. Consequently, promising extracts are ruled out because their activity disappears through fractionation [30].

The fact that these compounds show complete biological activity only when they interact in the crude extract [31,32] may be because that activity is conditioned not only on a clearly identified chemical structure, but also on a mixture of chemical structures that act together against a biological system such as a cell or an organism; in such a complex system of interactions, there very well may be multiple molecular targets [33]. These types of multiple interactions that explain why some extracts are more active than pure molecules are currently being studied through network pharmacology approximations [34,35]. This hypothesis suggests that although the biological activity of USBA 371 can be primarily attributed to DKPs, other compounds or chemical interactions are likely to contribute to its activity in the crude extract.

On the other hand, of the pure compounds we isolated, we were able to isolate a greater quantity of compound **1** than the others. Since compound **1** also demonstrated cytotoxic activity, we evaluated it against ten cell lines including murine and human tumor models and normal fibroblasts. Our results demonstrate the cytotoxic potential of compound **1** with high activity against the cell lines murine colon cancer MCA 38 (IC_50_ < 40 µM), breast cancer with high ALDH expression 4T1 H17 (IC_50_ < 40 µM) and lung cancer 3LL (IC_50_ < 51.71 µM) (Table 3). In contrast, no activity was detected against the myeloid acute leukemia K562 and U937 (Figure S11A–G) and we detected low activity, although not negligible, against the other cell lines analyzed (Table 3). 

Taking into consideration the complex dynamics of cancer and tumor models, it is necessary to evaluate the proliferation, metabolism, secretome and antigenicity of the tested cell lines. Here, compound **1** showed high selectivity for cancer cell lines, even against those displaying high drug resistance (Table 3). The highest cytotoxic activity was detected in the **3a** and **3b** mix of DKPs (cyclo (l-Phe-l-Pro) and cyclo (l-Ala-l-Pro), but the extracts yielded low quantities and this fraction was not used for further analyses. However, these results support the importance of studying metabolites with antitumoral activity in strain USBA 371. A common characteristic in the compounds here isolated is the presence of proline. It has been shown that DKPs containing proline such as cyclo (l-Pro-l-Tyr), cyclo (d-Pro-l-Phe), cyclo (d-Pro-l-Leu), cyclo (l-Pro-l-Met) and cyclo (l-Pro-d-Tyr) have biological activity against phytopathogenic fungi [37]. Extracts from *Pseudomonas aeruginosa* PAO1 containing a mix of DKPs cyclo (l-Pro-l-Tyr), cyclo (l-Pro-l-Val) and cyclo (l-Pro-l-Phe) showed activity against HeLa cervical carcinoma and Caco-2 colorectal adenocarcinoma cells (IC_50_ 0.53 and 0.66 mg/mL, respectively) [38]. The DKPs cyclo-(l-Val-l-Pro), cyclo-(l-Leu-l-Pro) and cyclo-(l-Phe-l-Pro) isolated from the endophytic strain *Streptomyces* SUK 25, have antimicrobial activity against methicillin-resistant *Staphylococcus aureus* and *Enterococcus raffinosus*, and low toxicity against HepaRG cell line from human hepatoma [23]. DKPs have also been isolated from microorganisms such as *Vibrio* sp., *Haloterrigena hispanica* and *Bacillus* sp. with antifungal and antibacterial activities [19,20,37]. DKPs cyclo (l-Leu-l-Pro) and cyclo (l-Phe-l-Pro) have shown activity against influenza A virus (H3N2) [39]. Iimura et al., (2017) showed that cyclo (l-Ala-l-Pro) inhibits the production of aflatoxines in the fungus *Aspergillus flavus* [40]. It has been shown that these DKPs play a role in different metabolic activities. In *Pseudomonas aeruginosa*, *Proteus mirabilis* and *Citrobacter freundii*, cyclo (l-Pro-l-Tyr) activates N-acylhomoserine lactones (AHLs) and it is also capable of activating or antagonizing the LuxR-based quorum-sensing systems [41]. 

This is the first report of DKPs isolated from the halophilic genus *Labrenzia* showing cytotoxic activity against human and murine tumor models. To shed light on the DKPs and secondary metabolite production potential of strain USBA 371, we sequenced and analyzed its genome for metabolic routes that could lead to DKPs and secondary metabolites production.

### 2.3. Secondary Metabolite Profiling, Genome Mining and Proteomics

The draft genome of *L. aggregrata* USBA 371 is composed of 6,417,675 bp with a calculated G + C content of 59.14%. It encodes 5916 protein coding genes, of which 4800 have a known function based on sequence similarity. We identified the 16S rRNA gene using the RNAmmer (v1.2) software and confirmed strain USBA 371 classification as *L. aggregata* with 99% similarity with the reference strain. The properties and statistics of the genome are summarized in Tables S1 and S2. The distribution of genes into clusters of orthologous groups (COGs) functional categories shows that most of them are involved in amino acid transport and metabolism (599, 10.2%), transcription (559, 9.5%) and carbohydrate transport and metabolism (527, 9.0%) categories (Figure S12). A total of 226 (3.8%) of the genes are involved in secondary metabolites biosynthesis, transport and catabolism.

The analysis for the presence of secondary metabolite biosynthetic gene clusters (BGCs) of ten strains belonging to four species of *Labrenzia* (*L. aggregata, L. alba, L. marina* and *L. suaedae*), including strain USBA 371, was performed using the IMG/MER platform. Our results reveal predominant clusters of NRPS genes, terpenes, bacteriocins, betalactones, *N*-acetylglutaminylglutamine amide (NAGGN) and type I PKS (Table 4).

In strain USBA 371, seven BGCs detected using antiSMASH [42] correspond to two NRPS, one type-I PKS, one NRPS-t1 PKS, one terpene, one bacteriocin and one betalactone. The bacteriocin, betalactone, terpene and TI PKS BGCs are not closely related to any known gene cluster, suggesting that novel BGC families could be discovered. The NRPS-T1PKS and NRPS show low sequence similarity (4% and 30%, respectively) to sporolide-nrps-t1pks and Turnerbactin, respectively (Table 5).

The cyclodipeptide synthases (CDPSs) are a family of enzymes that form a peptide bond between the amino acids of two aminoacyl-tRNAs (aa-tRNAs) that are used as substrate for formation of DKPs scaffolds [43]. We used the antiSMASH [42] and PRISM version 4.4.3 [44] tools for the discovery and classification of BGCs associated to CDPS [45] but failed to detect the presence of any CDPSs. A search of specific conserved domains related with domain/superfamily, functional sites and structural motif annotations for CDPS allowed the detection of 21,162 different domains of which none corresponded to CDPS. This would suggest that DKP production might be through NRPS enzymes, although the NRPSs detected in USBA 371 do not explain the production of DKPs reported in this study. 

**Figure 1:**
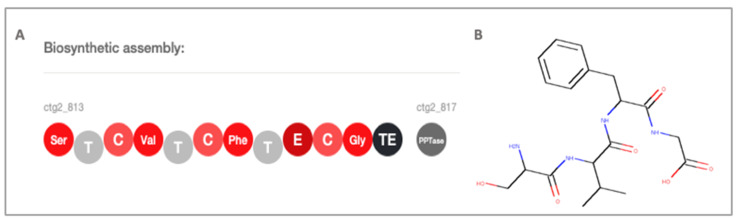
(**A**) PRISM version 4.4.3 prediction of the NRPS-T1PKS BGC, Ser: Serine adenylation domain, T: thiolation or peptidyl carrier protein, C: condensation, Val: Valine—adenylation domain, Phe: Phenylalanine—adenylation domain, E: epimerization, Gly: Glycine—adenylation domain, TE: thioesterase, PPTase: Phosphopantetheinyltransferase. (**B**) PRISM version 4.4.3 predicted cluster product.

The hybrid NRP-T1PKS corresponds to a BGC with four adenylation domains specific for the activation of serine, valine, phenylalanine and glycine according to PRISM version 4.4.3 annotation, while antiSMASH predicts the formation of the peptide nrp–valine–tyrosine–glycine. The BGC has thiolation, condensation, epimerization and thioesterase domains (Figure 1). 

Although the NRPSpredictor2 in the antiSMASH tool predicts with 100% certainty that two of the four adenylation domains have specificity for tyr and gly (Stachelhaus code match) amino acids, the substrates for the first two adenylation domains were predicted with a lower certainty (80%), suggesting that other amino acids than those predicted could be incorporated. Although the NRPS-T1PKS polypeptide chain cannot be associated with the diversity of DKPs found, it is possible that in case of iterative NRPS, multiple residues of the same amino acids are incorporated iteratively or in non-linear NRPS, which do not follow the domain organization, yielding unexpected products [46].

**Figure 2:**
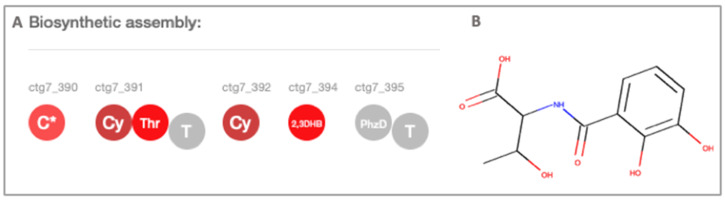
(**A**) PRISM version 4.4.3 prediction of the NRPS of strain USBA, C* or *Cy*: condensation domain, Thr: Threonine—adenylation domain, T: thiolation or peptidyl carrier protein; 2,3DHB: 2,3-dihydroxybenzoic acid, PhzD: Phenazine biosynthesis isochorismatase. (**B**) PRISM version 4.4.3 predicted cluster product.

The second NRPS identified has three condensation domains, two adenylation domains specific for threonine and 2,3-dihydroxybenzoic acid (2,3DHB) activation, one isochorismatase and one thiolation domain (Figure 2). Our proteomic analysis revealed peptides associated to isochorismatase and 2,3-dihydroxybenzoate-AMP ligase (Table S3), suggesting that they could be related to this BGC. We hypothesize that under our experimental conditions, some secondary metabolites may be produced in very low quantities making it difficult to isolate and identify them. 

Since we found no evidence for biosynthetic pathways associated with the CDPSs or NRPS, we suggest that they are produced by means of proteolytic enzymes such as dipeptidyl peptidases. They cleave the terminal ends of proteins to generate dipeptides which can then cyclize to form cyclodipeptides (CDPs) [47]. Our analysis identified five genes associated with the production of Xaa-Pro aminopeptidase and one gene associated with the production of Xaa-Pro dipeptidase. Proteomics analysis confirmed the bacterium’s production of endopeptidases and exopeptidases with 10 to 153 MS/MS spectral counts which suggests that they are very abundant in the four biological replicates analyzed (Table S3).

## 3. Materials and Methods

### 3.1. Accession Numbers

The draft genome of *Labrenzia aggregata* strain USBA 371 was generated at the DNA Sequencing Core Facility of Los Andes University (GenCore) following standard procedures. The *Labrenzia aggregata* USBA 371 draft Genome was deposited in the European Nucleotide Archive (ENA) under accession number ERS4291993 and the 16S rRNA gene sequence under accession number MF197928. 

### 3.2. Culturing Labrenzia aggregata USBA 371 to Obtain Metabolites in Crude Extracts from Chloroform Extraction

Cultures of strain USBA 371 were grown in TSB (Merck) 3% (*w*/*v*) NaCl in 0.5 L volumes during 48 h at 30° and 160 rpm. After cultures reached the stationary phase, they were centrifuged at 9000× *g* at 4 °C for 20 min to pellet the cells. The cell-free supernatants were extracted three times with chloroform (1:1 ratio) to obtain the chloroform crude extract. The organic phase was collected and evaporated to dryness under reduced pressure using a Buchi Rotavapor R114 (Buchi, Switzerland) and then stored at −20 °C in amber glass vials. Uninoculated culture medium was processed in the same way to obtain the medium extract here used as a negative control. Cultures and extractions were performed in three independent replicates.

### 3.3. Metabolic Profiling of the Crude Extract from Labrenzia aggregata USBA 371

Metabolic profiling of the chloroform crude extracts was performed using an HPLC system 1200 series coupled to Q-TOF 6520 (Agilent Technologies, Santa Clara, CA, USA). Chloroform extract of *Labrenzia aggregata* USBA 371 obtained as described above was used to inject 10 μL in a C18 column (Kinetex C18 150 mm × 2.1 mm, 2.6 μm; Phenomenex) with a guard column (Kinetex C18 20 mm × 2.1 mm, 2.6 μm; Phenomenex) as stationary phase. Chromatographic analysis was carried out at 40 °C using a gradient elution consisting of 0.1% (*v*/*v*) formic acid in water (A) and 0.1% (*v*/*v*) formic acid in acetonitrile (B) at a flow rate of 0.3 mL/min. The eluent gradient started from 25% B to 95% B in 35 min and was then kept constant for 10 min. Mass spectrometric detection was performed using electrospray ionization in positive mode in full scan, applying a mass range from 100 to 1200 *m*/*z*. The mass spectrometer source conditions consisted of a capillary voltage of 3500 V in positive mode, a scan rate of 1.02 scans per second. During all analysis, two reference masses were continuously injected for mass correction: *m*/*z* 121.0509 (C_5_H_4_N_4_) and *m*/*z* 922.0098 (C_18_H_18_O_6_N_3_P_3_F_24_). Features were putatively identified using the CEU Mass Mediator tool (http://ceumass.eps.uspceu.es/mediator/) [48] by matching the observed accurate mass of each compound with the *m*/*z* values available online using the four following databases: METLIN (http://metlin.scripps.edu), KEGG (http://genome.jp/kegg), lipid MAPS (http://lipidMAPS.org), and HMDB (http://hmdb.ca).

### 3.4. Isolation of Compounds from Crude Extract of L. aggregata USBA 371

The crude extract was fractionated by Sephadex LH20 using MeOH as solvent to obtain 8 fractions. Fractions 3 to 5 were pooled and then separated by preparative thin layer chromatography (p-TLC) using CH_2_Cl_2_-MeOH (9:1) to yield compounds **1** (15 mg) and **2** (2 mg). Fractions 6 to 8 were also pooled and separated by pTLC using the same discontinuous gradient of CH_2_Cl_2_-MeOH to yield the mixture of compounds **3a** and **3b** (4 mg). The identification of compounds **1**, **2**, **3a** and **3b** was done by NMR using a Bruker Fourier 300MHz. The 1D and 2D NMR data were collected, analyzed and the data were compared with the above-mentioned published in literature.

### 3.5. Cytotoxic Activity Assay of Major Compounds Isolated after Chloroform Extraction of Supernatants

The isolated compounds were resuspended in dimethyl sulfoxide (DMSO) to a final concentration of 25 mg/mL (stock solution), subsequent dilutions between 250 and 31 μg/mL were used for cytotoxicity assays. The cytotoxic activity was determined by neutral red assay [49], initially against the adherent cell lines 4T1 (mouse mammary tumor) and MCF-7 (human mammary adenocarcinoma) followed by H17 y TSA (murine breast cancer), MES SA PGP (+) y MES SA PGP (–) (human uterus sarcome), B16 (murine melanoma), MCA 38 (murine colon), 3T3 (murine fibroblats) y K562-ATCC, U937 y HL-60/MX2 (myeloid acute leukemia cell line) as described by Díaz-Cárdenas et al., 2017 [15]. When cultures reached a confluence of 80%, cells were passaged and counted in a Neubauer chamber. Cell viability was estimated in triplicate using the trypan blue assay. The percentage of viability was calculated as absorbance of the vehicle/absorbance of the treatment per 100. Inhibitory concentration 50 (IC_50_) values were defined as the concentration of the extracts that generated 50% inhibition of tumor cell growth. IC_50_ values were calculated with three or four parameter nonlinear regression curves using GraphPad Prism software (GraphPad Software, Inc., San Diego, CA, USA). IC_50_ values are presented as the mean ± standard error of mean (SEM). The selectivity index was calculated using the formula: IC_50_ Fibroblasts/IC_50_ tumor cell. A potential against tumor cell lines was considered when the selectivity was greater than 1.

The solvent used in the reconstitution of the extract (DMSO) was evaluated as a negative control for each test in the same volume as the extracts. Doxorubicin at a maximum concentration of 5 μM was used as a positive control.

### 3.6. Proteomic Analysis

Four independent cultures of strain USBA 371 were grown in TSB 3% (*w*/*v*) NaCl for 48 h at 30 °C and 160 rpm in darkness, and were marked as cultures a, b, c and d. The cultures were centrifuged at 6000× *g* for 30 min at 4 °C. Then, cells were transferred to 1.5 mL tubes and resuspended in an equal volume of Laemmli 2X buffer (Tris-HCl pH 6.8 65.8 mM, glycerol 26.3% (*v*/*v*), SDS 2.1% (*v*/*v*), bromophenol blue 0.01% and DTT 50 mM). The samples were heated at 99 °C for 5 min, sonicated for 5 min, and then subjected to bead-beating with a Precellys instrument (Bertin Technology, Montigny-le-Bretonneux, France) at 7800 rpm 3 times for 20 s. After a brief centrifugation at 16,000× *g* for 1 min, samples were diluted ½ in water. Proteins were then subjected to SDS-PAGE migration on a 4–12% NuPage gradient gel (Invitrogen) with MOPS buffer. After staining with Coomassie BlueSafe stain (Invitrogen), the corresponding polyacrylamide band containing the high molecular weight proteome (>200 kDa) of each sample was sliced, destained, treated with iodoacetamide, and proteolyzed in the presence of detergents to increase peptide recovery with trypsin as previously described [50]. The resulting peptides were resolved on a nanoLC reverse phase column connected to a high-resolution tandem mass spectrometer (Q-exactive HF instrument from Thermo Scientific, MA, USA) operated with a 60 min gradient in data-dependent acquisition mode. The parameters for MS and MS/MS acquisition were the same as previously described [51]. MS/MS spectra were interpreted against the protein sequence database specific of strain USBA 371 with the MASCOT software (Matrix Science) as described [51]. A list of the identified proteins was established (false discovery rate (FDR) below 1% as assessed with a decoy search), and their quantities were evaluated based on their spectral counts. 

### 3.7. Genome Sequencing, Assembly and Annotation

*Labrenzia aggregata* USBA 371 was grown on 5 mL of Marine Medium 2216 (Difco) at 30 °C for 24 h and 160 rpm. Cells were harvested by centrifugation at 4000 rpm when the mid exponential phase was reached, pelleted and immediately used for DNA extraction. We extracted the genomic DNA using the Wizard^®^ Genomic DNA Purification Kit (Promega) according to the manufacturer’s instructions. Illumina 250PE insert standard shotgun library was designed and sequenced using an Illumina HiSeq. Raw reads were analyzed using FastQC (v0.11.2) (http://www.bioinformatics.bbsrc.ac.uk/projects/fastqc/), filtering of data (phred score <30, read length <80bp and PCR-sequencing adapters) was performed using Trimmomatic (v0.36) [52]. Remaining reads were assembled using SPADES (v3.9) [53].

Due to an oversized assembly (10 Mbp against 6.5 Mbp of the reference *L. aggregata* genome, accesion number NZ_CP019630.1), filtered reads were processed and re-assembled after running a Blastn against nt database. Genes were identified using the U.S. Department of Energy (DOE) Joint Genome Institute (JGI) (DOE-JGI) genome annotation pipeline [54], and functional annotation was performed within the Integrated Microbial Genomes (IMG) platform [54] and RASTtk [55] protocol. The USBA 371 annotated Genome was uploaded to the GOLD database under the Integrated Microbial Genomes (IMG) ID accession number 2827789216. The assembled genome was processed for secondary metabolites detection using web tool antiSMASH (v2.0) [42] (http://antismash.secondarymetabolites.org/), and PRISM version 4.4.3 [44]. A search of specific conserved domains related with domain/superfamily, functional sites and structural motif annotations for CDPs and NRPs in JGI-annotated genome was performed through rpsBlastn against the conserved domain database (CDD) (https://www.ncbi.nlm.nih.gov/cdd/). Output matches were processed through rpsbproc utility using an E-value of 0.01 and a concise level of redundancy of domain hit.

The exploration of microbial secondary metabolism in *Labrenzia* spp., were done using the Integrated Microbial Genomes Atlas of Biosynthetic gene Clusters (IMG-ABC) (https://img.jgi.doe.gov/abc-public), a publicly available database of predicted biosynthetic gene clusters [56].

## 4. Conclusion and Perspectives

We show that USBA 371 extracts contain bioactive metabolites and DKPs with cytotoxic activity against human and murine tumor models. This study paves the way for evaluating the pharmacological properties and therapeutic potential of metabolites identified in USBA 371. Since levels of biological activity of crude extracts are higher than those of the compounds that were isolated, it remains to be determined whether the use of complex mixtures of bacterial extracts that present biological activities with high therapeutic potential is feasible. If it is, it would open an interesting path in the search for active molecular complexes beyond isolated compounds which could become an innovative vision in the search for antitumor drugs.

In addition, the introduction of the *Labrenzia* genus to bioprospecting is likely to lead to the identification of other secondary metabolites, especially considering that information from the BGCs that we have identified has opened a new line of research that is likely lead to the discovery of new metabolites. 

## Figures and Tables

**Table 1:** Metabolic profile of crude extract from strain USBA 371 by LC-MS analysis (*n* = 3).

Compound	Molecular Formula	Molecular Weight (DB) g/mol	Mass Error (ppm)	Observed Ion	Description
Peptides					
*Cyclo* (l-leucyl-l-phenylalanyl)	C_15_H_20_N_2_O_2_	260.1525	3	M + H	Cyclic dipeptides or diketopiperazines (DKPs) are a large class of natural products with biological activities. Dipeptide and tripeptides are incomplete breakdown product of protein digestion or protein catabolism.
*Cyclo* (l-leucyl-l-leucyl)	C_12_H_22_N_2_O_2_	226.1681	4	M + H	
*Cyclo* (l-Pro-l-Tyr)	C_14_H_16_N_2_O_3_	260.151	1	M + H	
*Cyclo* (l-Ala-l-Pro)	C_8_H_12_N_2_O_2_	168.07	1	M + H	
Aspartyl-Lysine//Gly Val Ser	C_10_H_19_N_3_O_5_	261.1325	3	M + Na	
Arg Gln Gln	C_16_H_30_N_8_O_6_	430.2288	8	M + Na	
Lysyl-Asparagine	C_10_H_20_N_4_O_4_	260.1485	6	M + H	
Tyr Trp Ile	C_26_H_32_N_4_O_5_	480.2373	3	M + H	
His Phe Trp	C_26_H_28_N_6_O_4_	488.2172	4	M + H	
Trp Tyr Phe	C_29_H_30_N_4_O_5_	514.2216	4	M + H	
Alkaloids					
Anthcolorin G	C_33_H_47_NO_4_	521.7305	1	M + H	
Fatty Acyls					
Dodecanamide	C_12_H_25_NO	199.1936	1	M + H	Fatty acids comprise components of the dual-membrane envelope in bacteria.
Linoleic acid	C_18_H_32_O_2_	280.2402	4	M + H	
α-Linolenic acid	C_18_H_30_O_2_	278.2246	4	M + H	
*N*-palmitoyl isoleucine//Arachidoyl glycine	C_22_H_43_NO_3_	369.3243	5	M + H	
Oleamide	C_18_H_35_N_O_	281.2719	4	M + H	
Palmitic acid	C_16_H_32_O_2_	256.2402	5	M + H	
Oleic acid	C_18_H_34_O_2_	282.2559	3	M + H	
*N*-Butyl arachidonoyl amine	C_24_H_41_NO	359.3188	4	M + H	
Tetradecenoyl-CoA	C_35_H_60_N_7_O_17_P_3_S	975.2979	2	M + Na	
Glycerolipids					
Monoacylglyceride (16:0)	C_19_H_38_O_4_	330.277	5	M + H	Glycerolipids are complex lipids formed by the condensation of one, two, or three fatty acid molecules on glycerol.
Diglyceride (44:6)	C_47_H_80_O_5_	724.6006	6	M + Na	
Glycerophospholipids					
Lysophosphatidylcholine (LPC) (16:1)//Phosphatidylethanolamine (PE) (19:1)	C_24_H_48_NO_7_P	493.3168	4	M + H	Glycerophospholipids comprise components of the dual-membrane envelope of Gram-negative bacteria with biological functions in protein binding, transport of proteins across inner membranes.
Lysophosphatidylcholine (O-18:0)	C_26_H_52_NO_7_P	521.3481	4	M + H	
Phosphatidylethanolamine (39:5)	C_44_H_78_NO_8_P	779.5465	2	M + H	
Phosphatidylethanolamine (41:6)	C_46_H_80_NO_8_P	805.5622	2	M + H	
Phosphatidylethanolamine (39:4)	C_44_H_80_NO_8_P	781.5622	6	M + H	
Phosphatidylethanolamine (41:5)	C_46_H_82_NO_8_P	807.5778	1	M + H	
Phosphatidylglycerol (31:3)	C_37_H_67_O_10_P	702.4471	5	M + H-H20	

**Table 2:** Cytotoxic activity of crude extract from chloroform extraction (USBA 371 cultured in TSB 3% (*w*/*v*) NaCl) and the isolated compounds against cell lines MCF-7 and 4T1.

	Cytotoxic Activity IC_50_	
Strain/Fraction	Cellular Line	
	MCF-7	4T1
Strain USBA 371	4.5 ± 1.3 µg/mL	5.5 ± 3.44 µg/mL
Compound **1**	87.74 ± 2.32	57.09 ± 2.11 µM
Compound **2**	890.53 ± 3.45 µM	875.93 ± 5.14 µM
Compound mix **3a** and **3b**	16.10 ± 1.66 µg/mL	13.41 ± 1.64 µg/mL

**Table 3:** Cytotoxic activity of compound **1** against tumor cell models.

Cell Line		Cytotoxic Activity of Fractions IC_50_ (µM)	Selectivity Index (SI)
		Compound 1	
Murine Breast Cancer	4T1 H17 *	40.38 ± 1.94	7.78
	TSA	196.66 ± 4.18	1.59
Murine Melanoma	B16	80.87 ± 3.67	3.88
Murine Colon	MCA 38	29.85 ± 1.55	10.52
Human Uterus Sarcome	MES–SA/DX5P-Pgp (+)	225.28 ± 1.23	1.39
	MES–SA/DX5P-Ppg (−)	91.32 ± 5.64	3.44
Lung	3LL	51.71 ± 0.55	6.07
Myeloid Acute Leukemia	K562	ND	ND
	U937	ND	ND
Murine Fibroblast	3T3	314.30 ± 3.41	ND

Selectivity index indicates fractions toxic to tumor model compared to healthy cells (>1) or vice versa (<1). ND: Not detected. * Murine tumor cell line with high ALDH+ expression and high chemotherapy resistance [36].

**Table 4:** Number and type of biosynthetic gene cluster groups obtained from 10 strains of *Labrenzia* genus.

Biosynthetic Gene Cluster (BGC) Type *	*Labrenzia Aggregata* Strain USBA 371	*Labrenzia Aggregata* CECT 4801	*Labrenzia Aggregata* LZB033	*Labrenzia Marina* DSM 17023	*Labrenzia Suaedae* DSM 22153	*Labrenzia Alba* CECT 5096	*Labrenzia Alba* CECT 7551	*Labrenzia Alba* VG12	*Labrenzia Alba* CECT 5095	*Labrenzia Alba* CECT 5094
Betalactone	1	1	1	2	2	1	1	1	1	1
Bacteriocin	1	1	1	1	2	1	1	1	1	1
Ectoine, Hserlactone	0	0	0	0	0	0	0	0	0	0
NAGGN	1	1	1	1	1	0	0	1	0	0
T1PKS	1	1	1	1	1	0	1	1	0	0
Terpene	1	1	1	1	1	1	1	1	1	1
NRPS	2	4	6	1	1	0	0	1	0	0
TransAT-PKS, T3PKS-NRPS	0	0	0	0	1	1	0	0	1	1
Thiopeptide	0	0	0	0	0	1	0	0	1	1
NRPS-T1PKS	1	0	0	1	0	0	0	0	0	0
TOTAL BGC	8	9	11	8	8	5	4	6	5	5

* Cluster types according to Integrated Microbial Genomes Atlas of Biosynthetic gene Clusters (IMG-ABC) (https://img.jgi.doe.gov/abc-public).

**Table 5:** Biosynthetic gene cluster information. Summary of the antiSMASH biosynthesis cluster prediction in the genome of *L. aggregata* USBA 371.

Biosynthetic Gene Cluster (BGC) Type	From	To	Most Similar Known Cluster	Similarity	MIBiG BGC-ID
NRPS-T1PKS	872,702	926,444	Sporolide-nrps-t1pks	4%	BGC0000150
T1PKS	963,299	1,010,906	ND		
Bacteriocin	125,918	136,814	ND		
Betalactone	82,927	105,536	ND		
Terpene	492,697	513,548	ND		
NRPS	405,475	443,932	Turnerbactin	30%	BGC0000451

## Supplementary Materials

The Supplementary Materials are available online at https://www.mdpi.com/1420-3049/25/11/2546/s1, Figure S1: 1H-NMR for compound **1** (CDCl_3_, 300 MHz), Figure S2: ^13^C-NMR for compound **1** (CDCl_3_, 75 MHz), Figure S3: HSQC spectrum for compound **1** (CDCl_3_, 300 MHz), Figure S4: COSY H-H spectrum for compound **1** (CDCl_3_, 300 MHz), Figure S5: HMBC spectrum for compound **1** (CDCl_3_, 300 MHz), Figure S6: ^1^H-NMR for compound **2** (CDCl_3_, 300 MHz), Figure S7: ^13^C-NMR for compound **2** (CDCl_3_, 75 MHz), Figure S8: ^1^H-NMR for the mixture of compounds **3a** and **3b** (CDCl_3_, 300 MHz), Figure S9: ^13^C-NMR for the mixture of compounds **3a** and **3b** (CDCl_3_, 75 MHz), Figure S10: The cytotoxic activity of the crude extract, compounds **1** and **2**, and the mix of compounds **3a** and **3b** assessed against cell lines 4T1 (A) and MCF-7 (B), Figure S11: The cytotoxic activity of compound **1** against (A) Murine melanoma cell line B16; (B) murine colon cell line MCA 38; (C) Human uterus sarcoma cell line; (D) Lung cancer cell line 3LL; (E) Murine fibroblast cell line 3T3; (F) Myeloid acute leukemia cell line K562; (G) Myeloid acute leukemia cell line U937, Figure S12: Gene categories associated with general COG functional categories in *Labrenzia aggregata* strain USBA 371, Table S1: Quast statistics for assembly, Table S2: Genome statistics of *Labrenzia aggregata* USBA371, Table S3: List of proteins detected in *Labrenzia aggregata* USBA 371 using proteomic analysis.
