# Supplementary material for: Bioactive Potential of Extracts of Labrenzia aggregata Strain USBA 371, a Halophilic Bacterium Isolated from a Terrestrial Source
Source: Molecules. 2020 May 29;25(11):2546. doi: 10.3390/molecules25112546 (PMC7321072; doi:10.3390/molecules25112546)
Supplement: Supplementary file 1 [file molecules-25-02546-s001.zip › molecules-771590-supplementary-proof revised/Supplementary Material.pdf]

Supplementary material

Bioactive potential of extracts of *Labrenzia aggregata* strain USBA 371, a halophilic bacterium isolated from a Terrestrial source

Carolina Díaz-Cárdenas [1], Laura Yinneth Rojas [2], Susana Fiorentino [2], Monica P. Cala [3], Jorge I Díaz [4], Freddy A. Ramos [5], Jean Armengaud [6], Silvia Restrepo [7] and Sandra Baena [1,*]

[1] Unidad de Saneamiento y Biotecnología Ambiental, Departamento de Biología, Pontificia Universidad Javeriana, P.O. Box 56710 Bogotá DC, Colombia; diazcardenascaro@gmail.com

[2] Grupo de Inmunobiología y Biología Celular. Pontificia Universidad Javeriana, P.O. Box 56710 Bogotá DC, Colombia; rojasl.a@javeriana.edu.co (L.Y.R.); susana.fiorentino@javeriana.edu.co (S.F.)

[3] Metabolomics Core Facility—MetCore, Vicepresidency for Research and Creation, Universidad de los Andes, Cra 1 No. 18A-12, 111711 Bogotá DC, Colombia; mp.cala10@uniandes.edu.co

[4] Vicepresidency of Research and Creation, Universidad de los Andes, Cra 1 No. 18A-12, 111711 Bogotá DC, Colombia; ji.diaz1@uniandes.edu.co

[5] Departamento de Química, Facultad de Ciencias, Universidad Nacional de Colombia-Sede Bogotá, Carrera 30 # 45-03, 110111 Bogotá DC, Colombia; faramosr@unal.edu.co

[6] Département Médicaments et Technologies pour la Santé (DMTS), CEA, INRAE, Université Paris Saclay, SPI, 30200 Bagnols-sur-Cèze, France; jean.armengaud@cea.fr

[7] Chemical Engineering Department, Universidad de los Andes, Cra 1 No. 18A-12, 111711 Bogotá DC, Colombia; srestrep@uniandes.edu.co

* Correspondence: baena@javeriana.edu.co

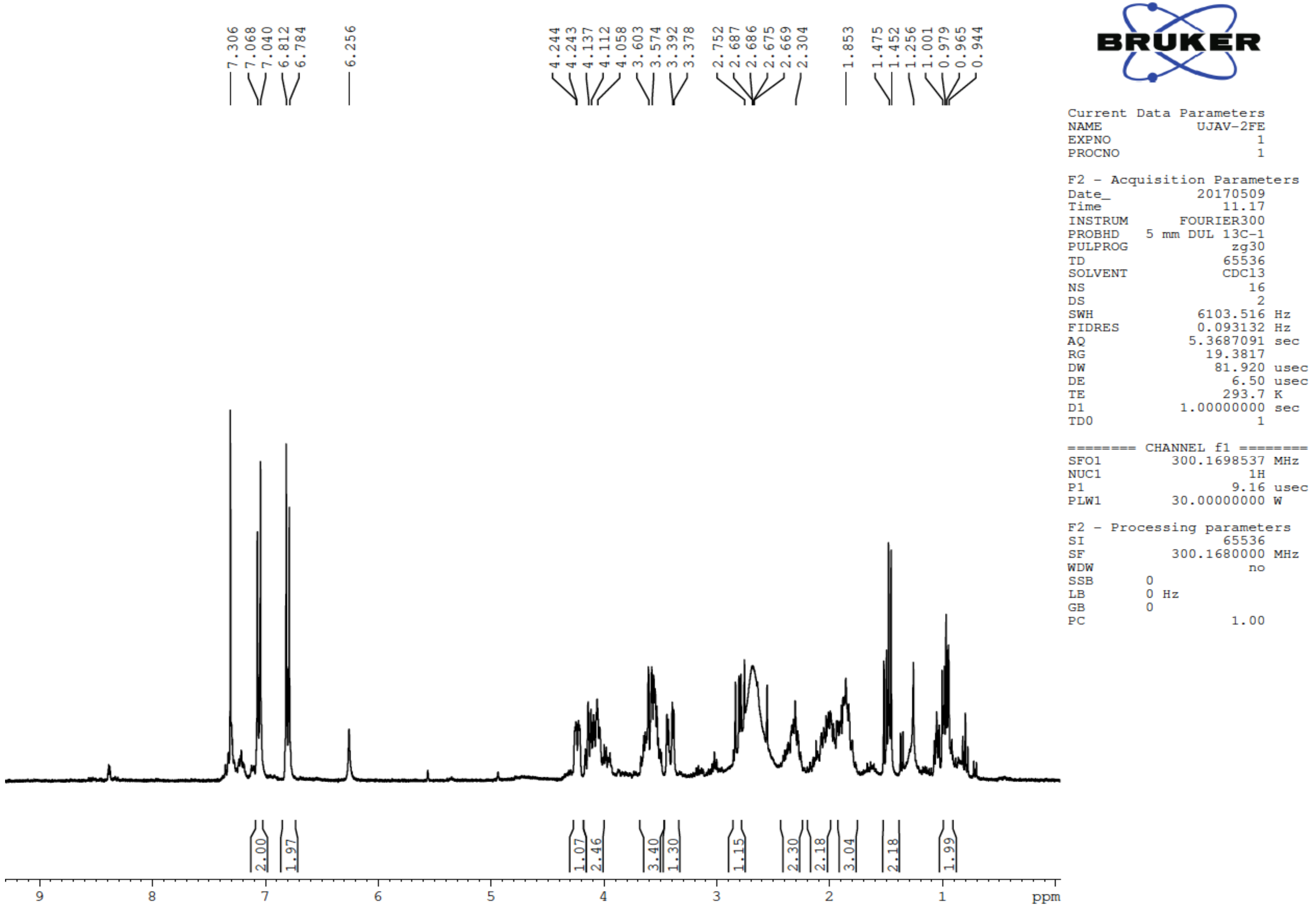

Supplementary Figure S1. ^{1}H-NMR for compound 1 ($CDCl_3$, 300 MHz)

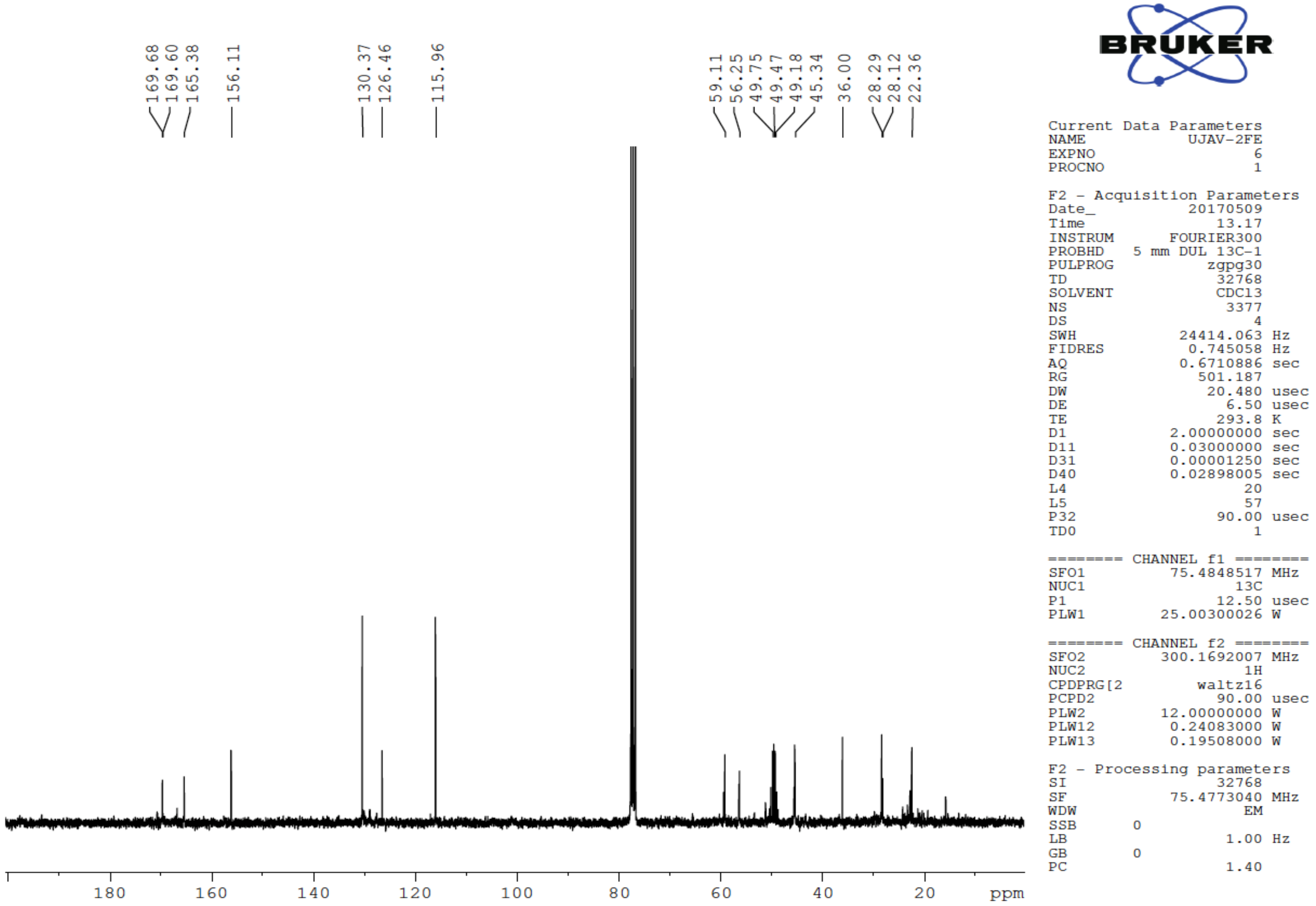

Supplementary Figure S2. ^{13}C-NMR for compound 1 ($CDCl_3$, 75 MHz).

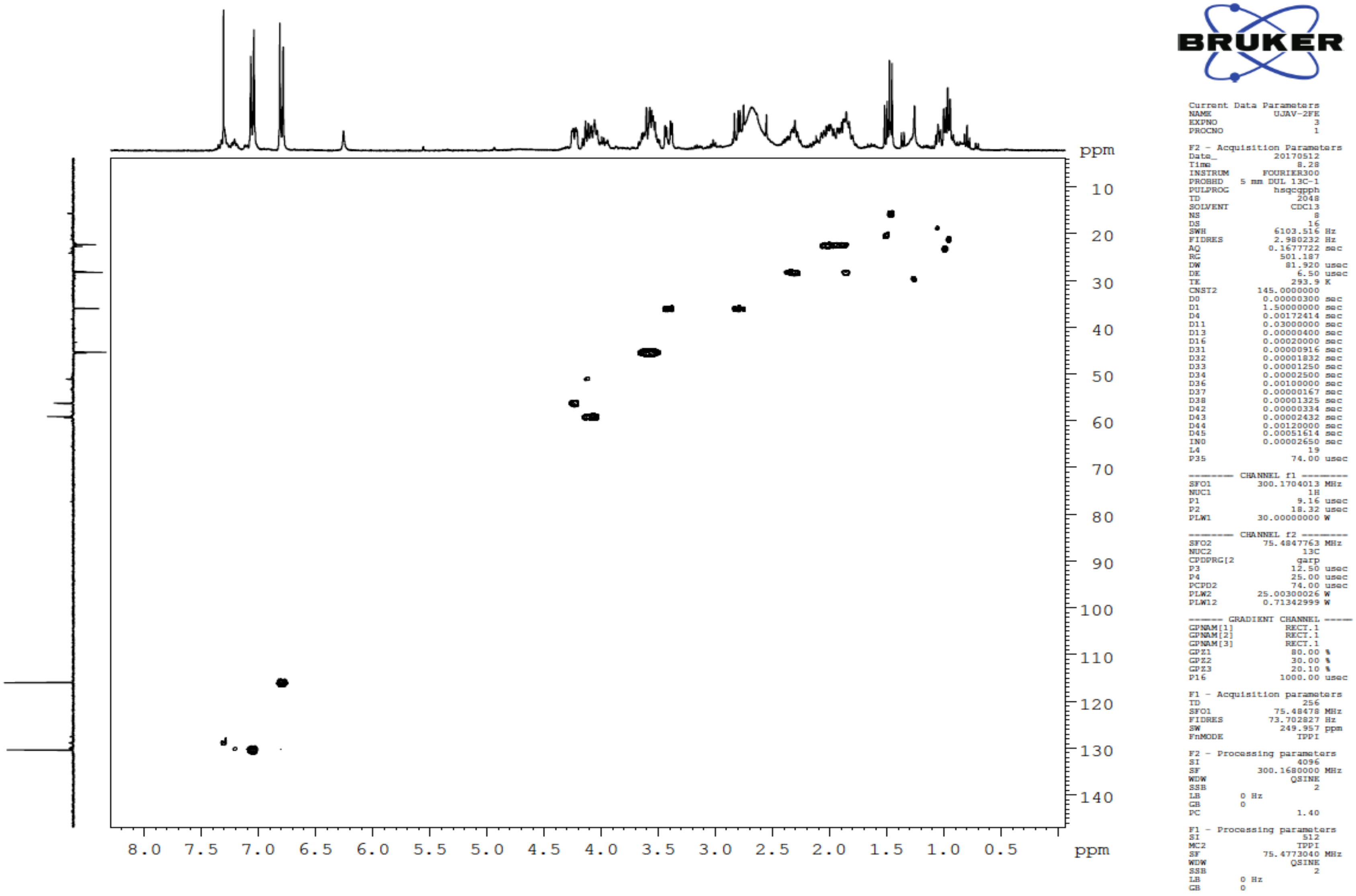

Supplementary Figure S3. HSQC spectrum for compound 1 ($CDCl_3$, 300 MHz).

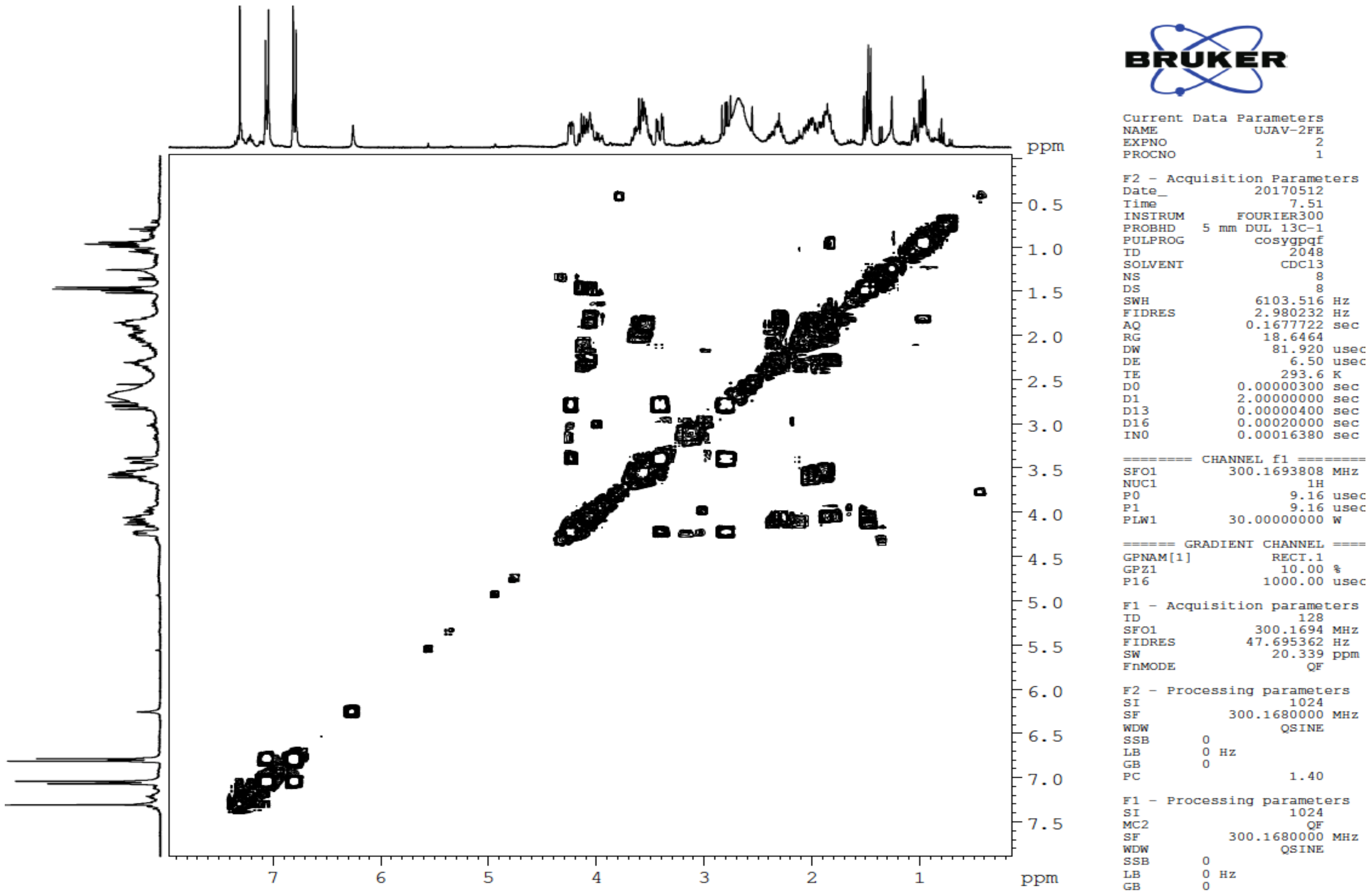

Supplementary Figure S4. COSY H-H spectrum for compound 1 ($CDCl_3$, 300 MHz).

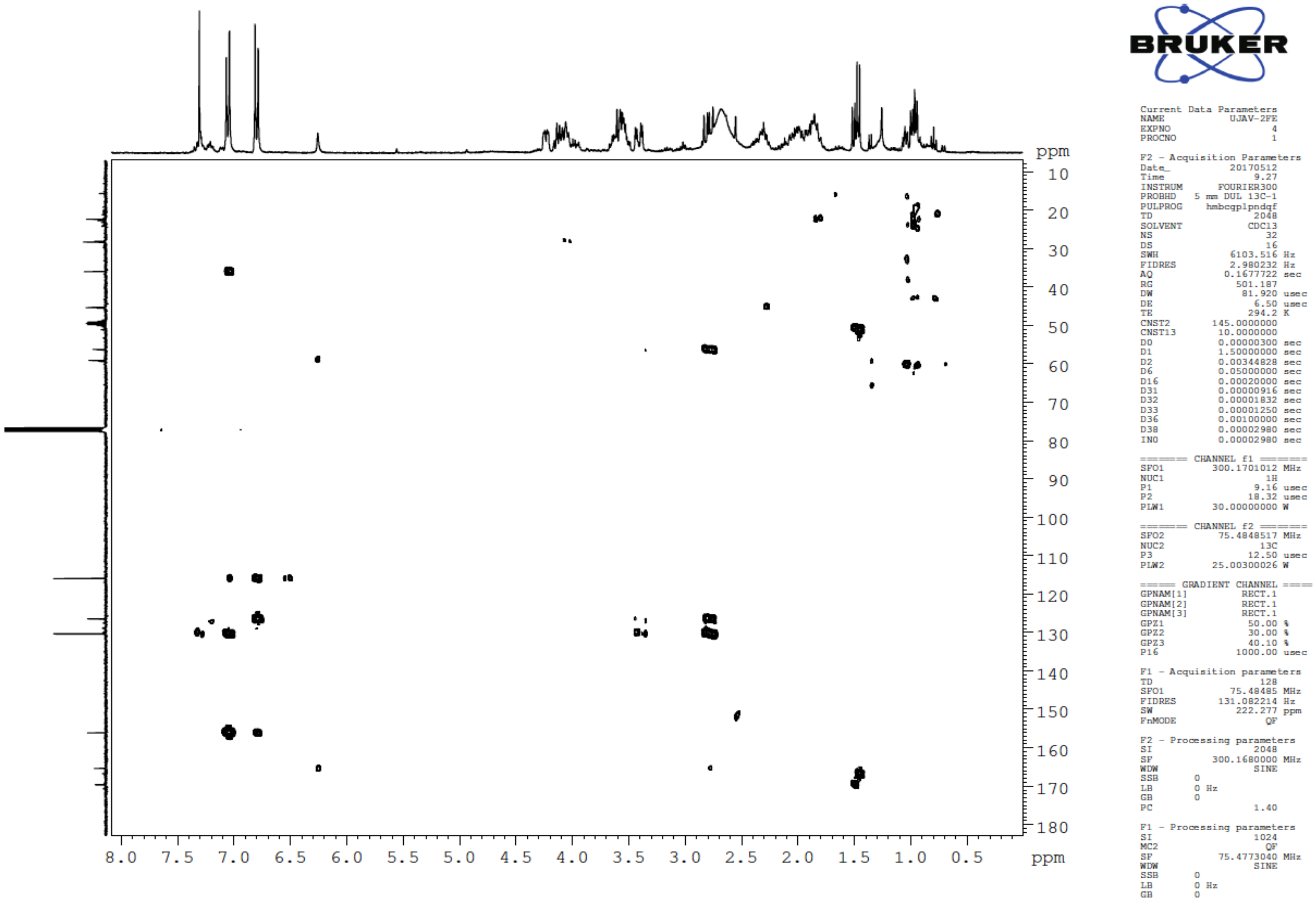

Supplementary Figure S5. HMBC spectrum for compound 1 ($CDCl_3$, 300 MHz).

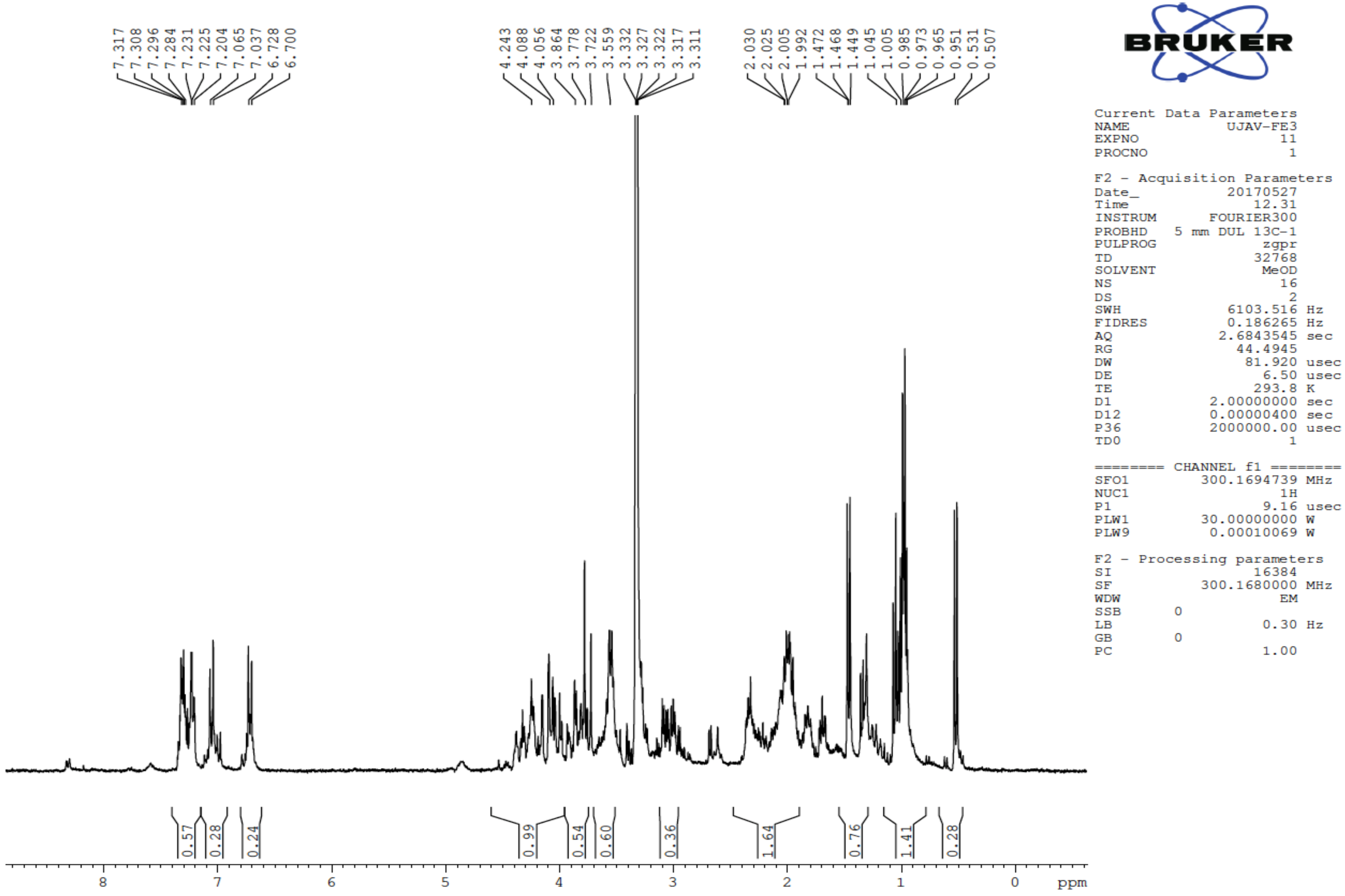

Supplementary Figure S6. ^{1}H-NMR for compound 2 ($CDCl_3$, 300 MHz)

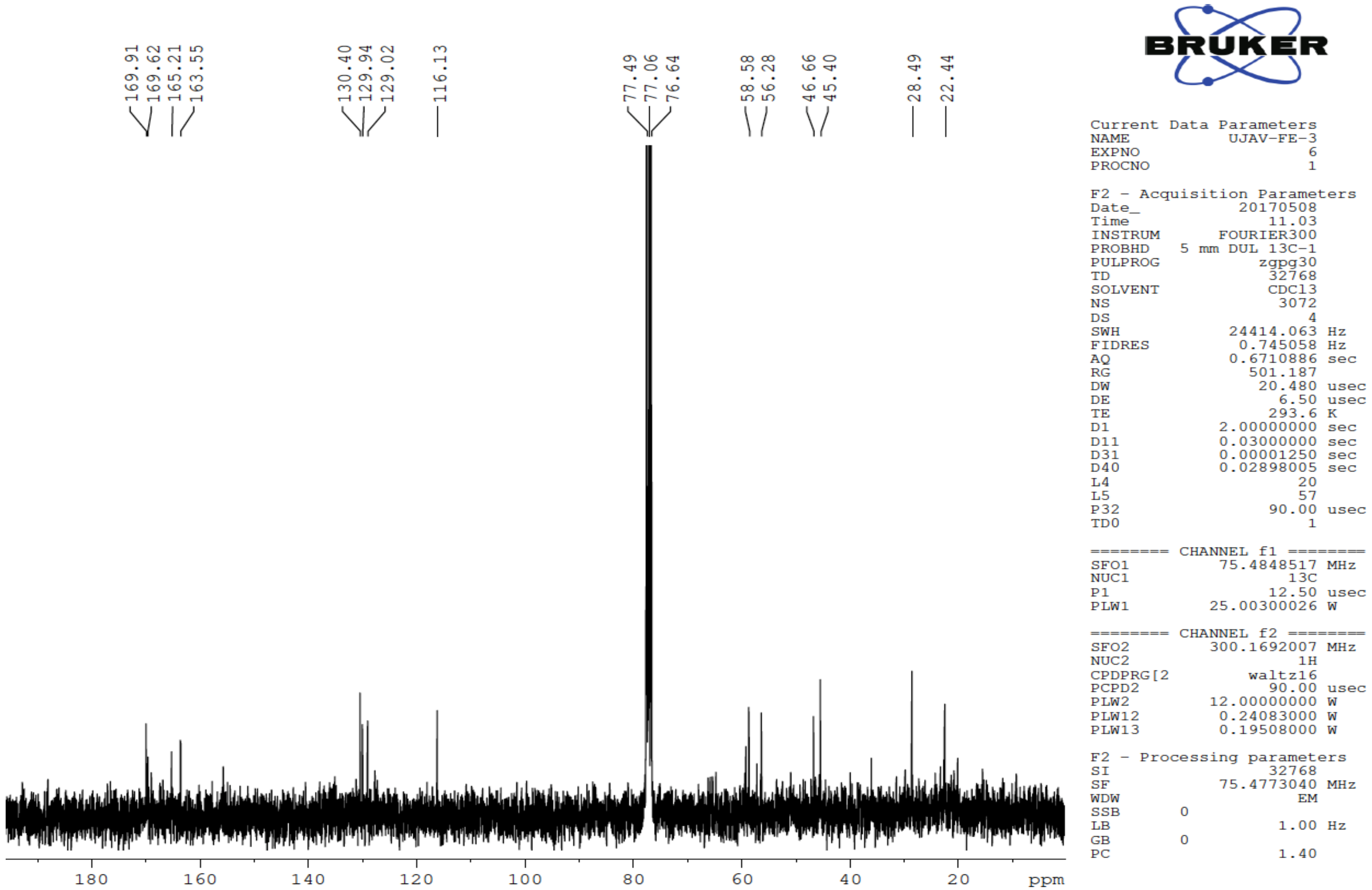

Supplementary Figure S7. ^{13}C-NMR for compound 2 ($CDCl_3$, 75 MHz)

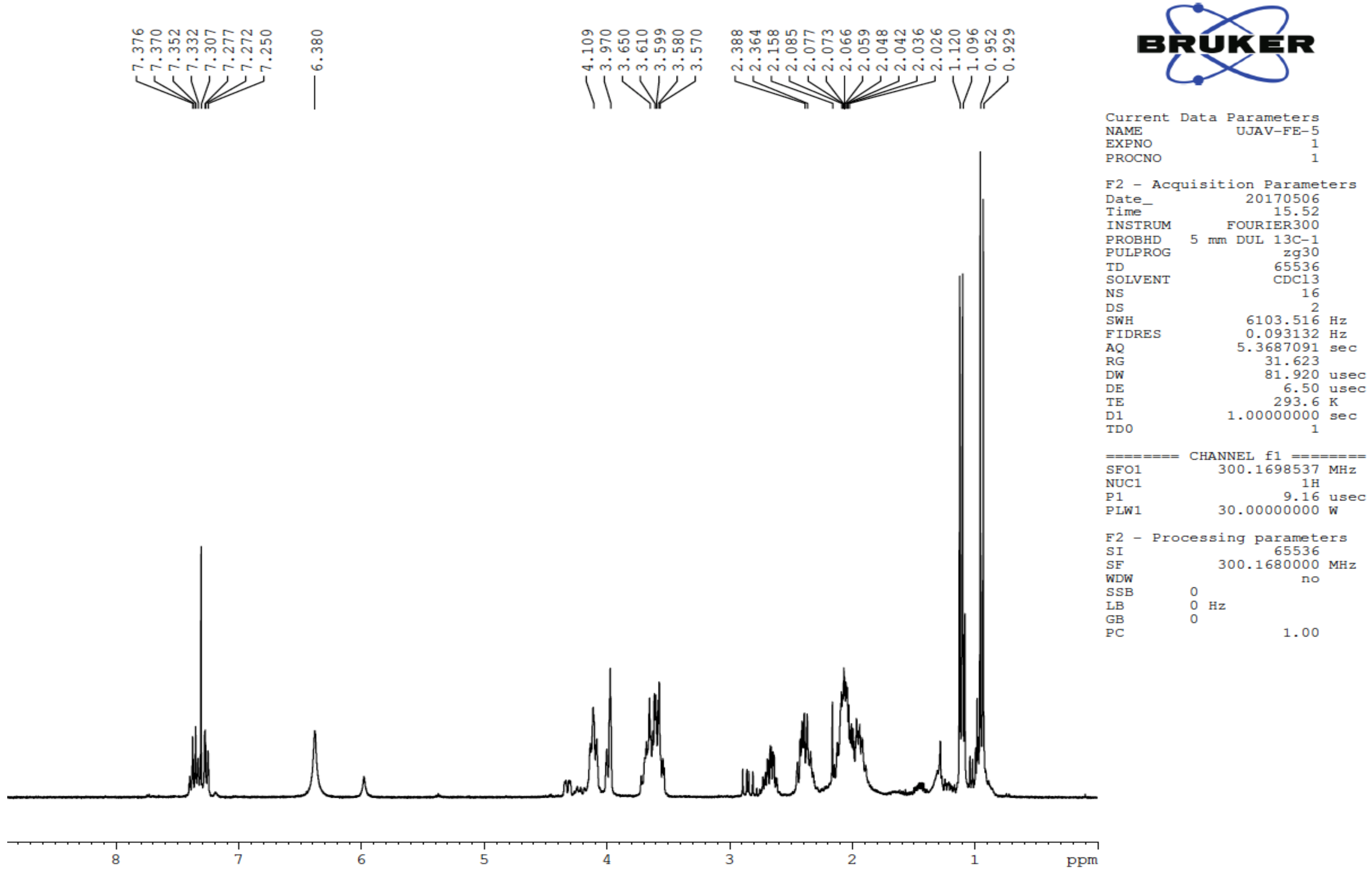

Supplementary Figure S8. ^{1}H-NMR for the mixture of compounds 3a and ·3b ($CDCl_3$, 300 MHz).

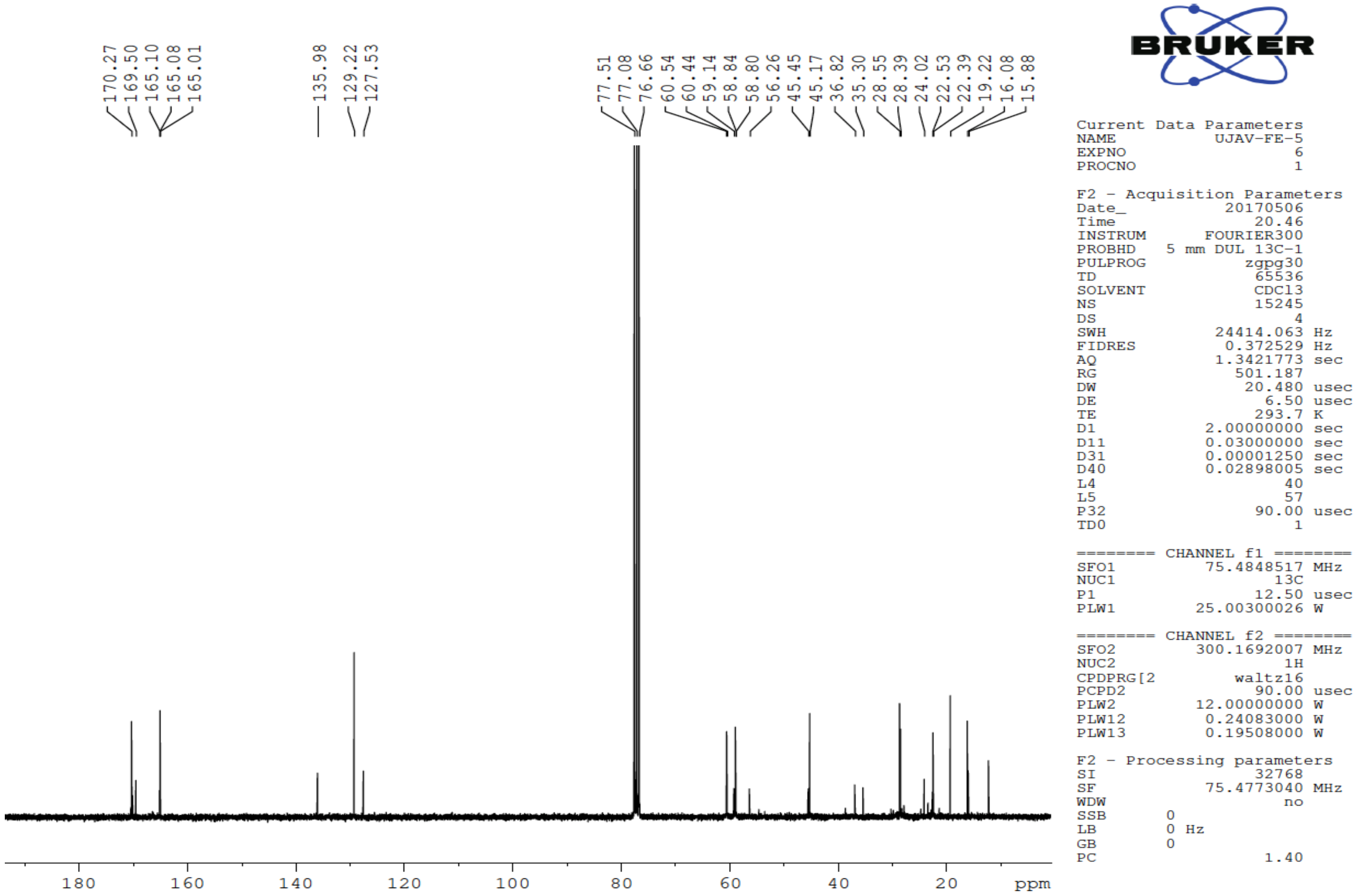

Supplementary Figure S9. ^{13}C-NMR for the mixture of compounds 3a and ·3b ($CDCl_3$, 75 MHz)

A

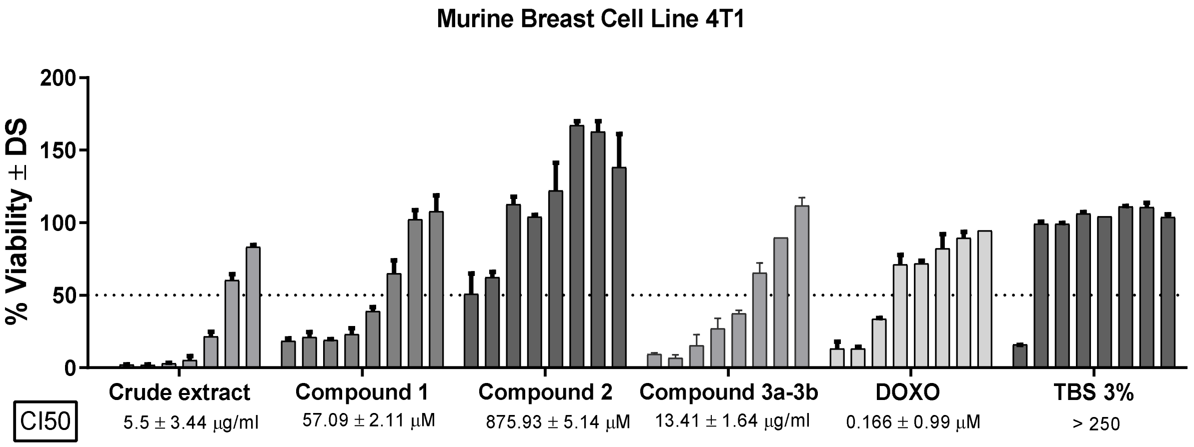

B

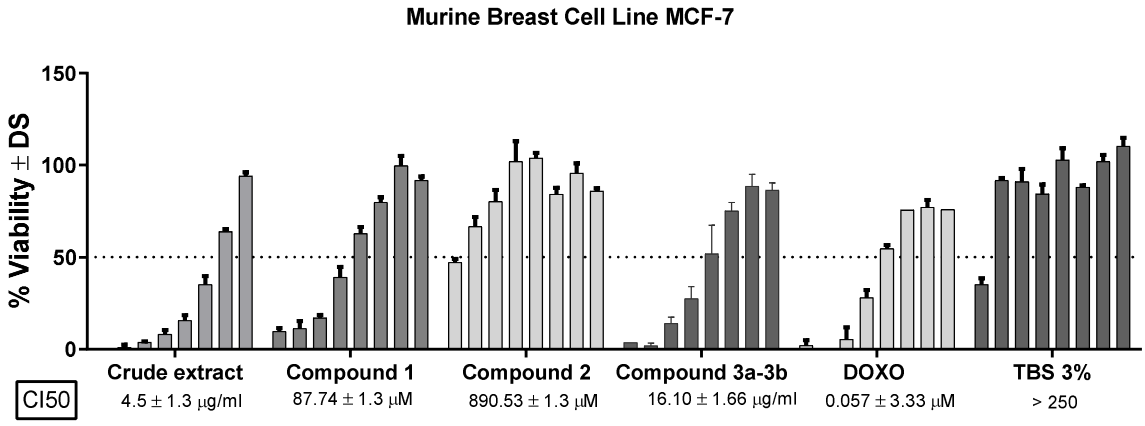

Supplementary Figure S10. The cytotoxic activity of the crude extract (µg/mL), compounds 1 (µM) and 2 (µM), and the mix of compounds 3a and 3b (µg/mL), was assessed against cell lines 4T1(A) and MCF-7 8B). The extract from uninoculated TSB 3 % (w/v) NaCl was used as control.

A

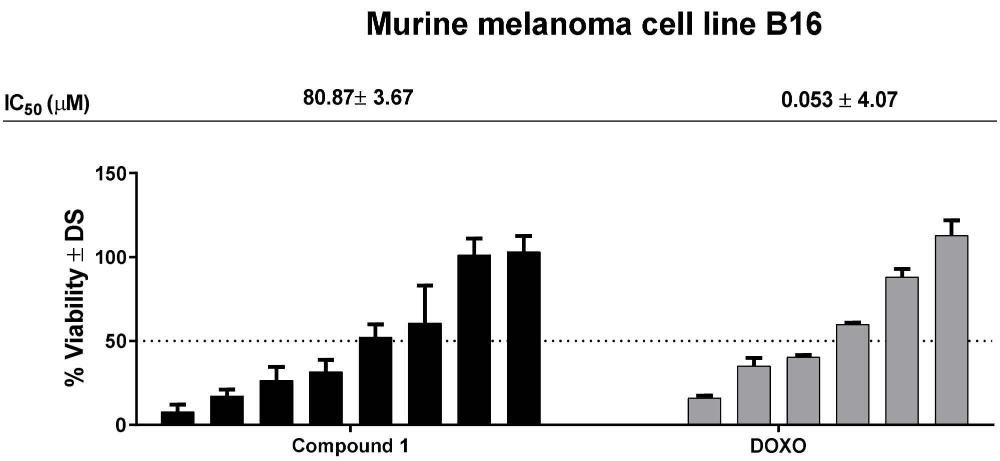

B murine colon cell line MCA 38

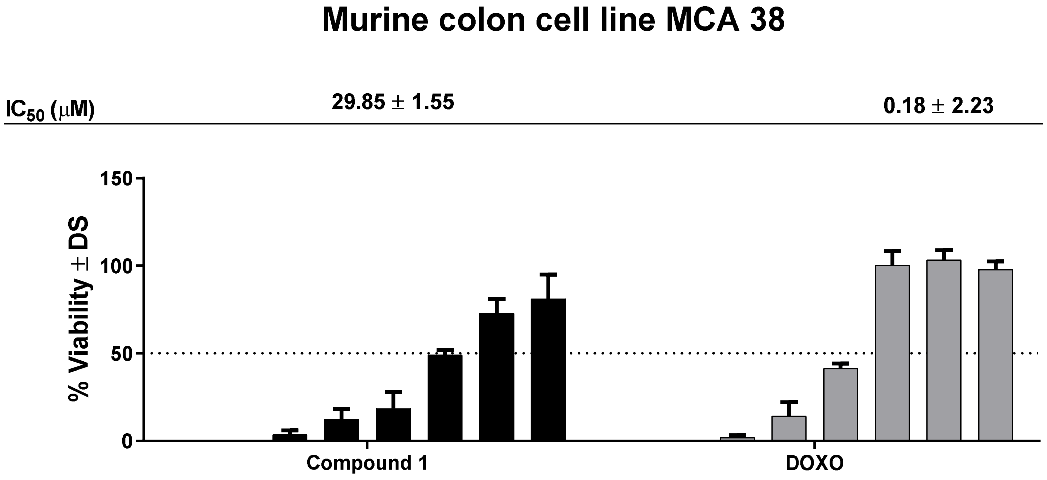

C.

Human uterus sarcome cell line

IC_{50} (μM)	225.28 ± 1.23	2.15 ± 1.43	91.32 ± 5.64	2.56 ± 2.85

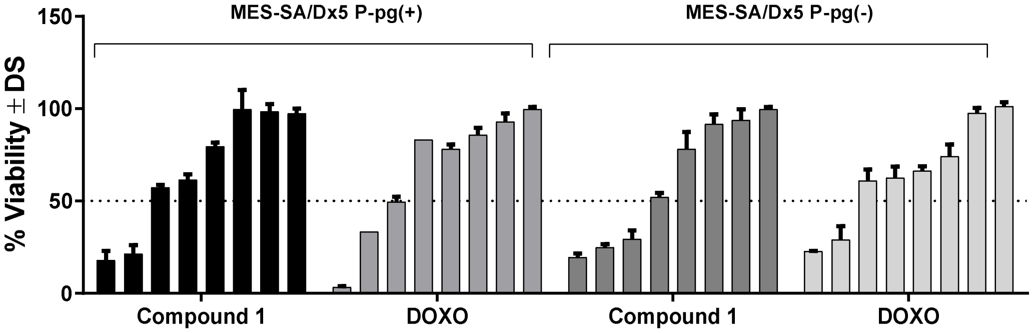

D.

Lung cancer cell line 3LL

IC_{50} (μM)	51.71 ± 0.55	0.049 ± 1.22

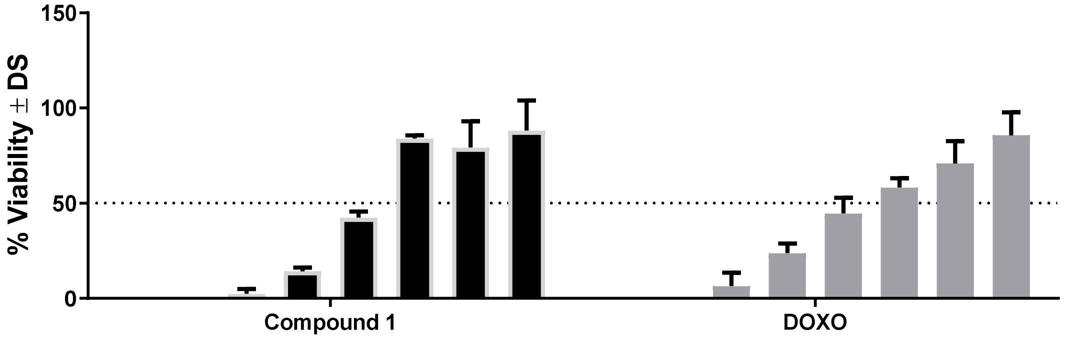

E.

Murine fibroblast cell line 3T3

IC_{50} (µM)	314.30 ± 3.41	0.018 ± 5.02

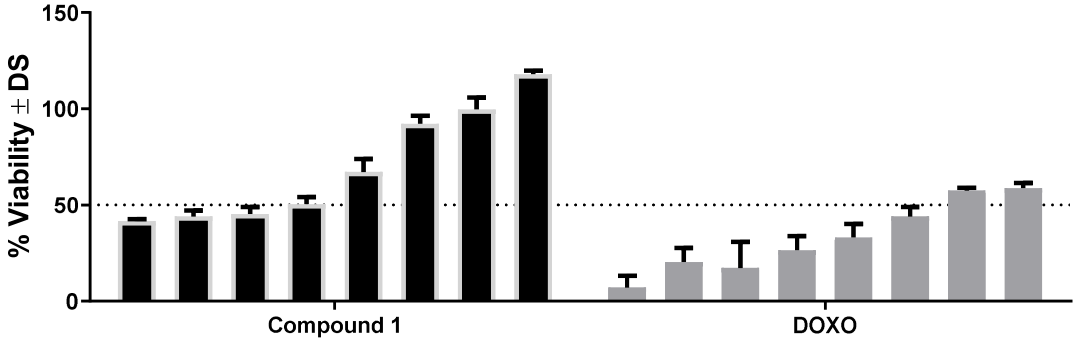

F.

Myeloid Acute Leukemia Cell Line K562

IC_{50} (µM)	NC	1,816 ± 2.34

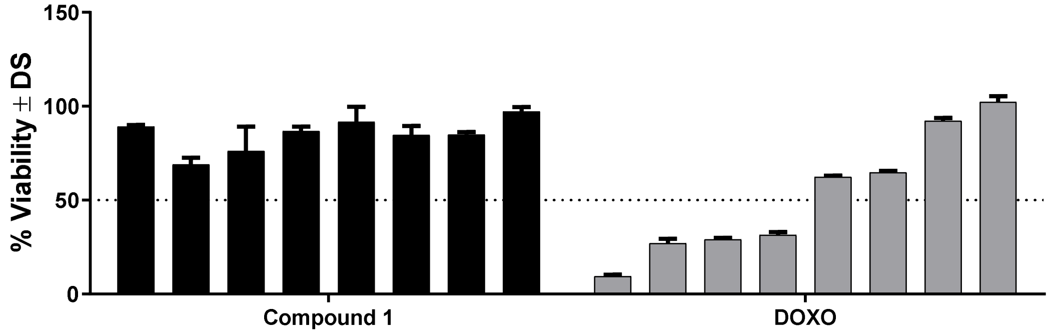

G.

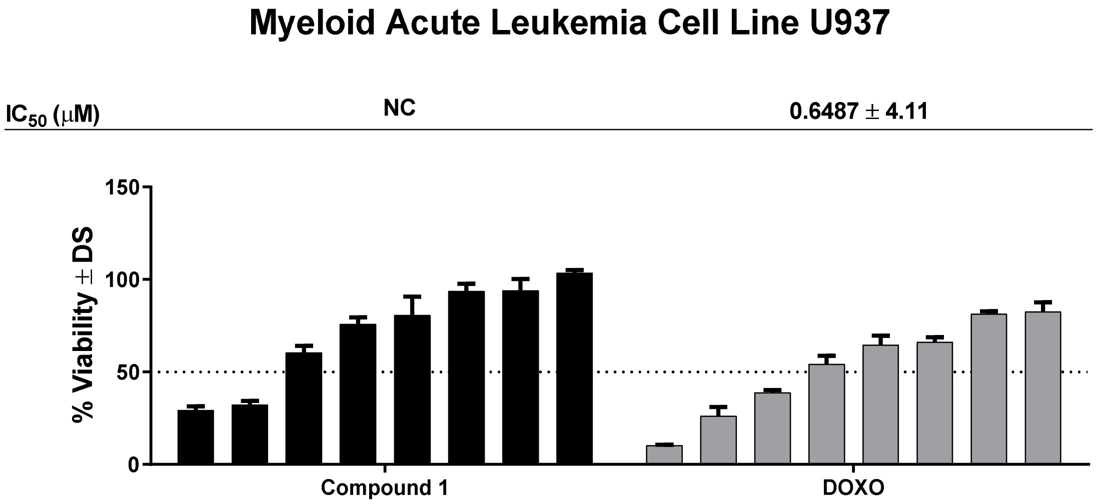

Supplementary Figure S11. The cytotoxic activity of compound **1** against A) Murine melanoma cell line B16; B) murine colon cell line MCA 38; C) Human uterus sarcoma cell line; D) Lung cancer cell line 3LL; E) Murine fibroblast cell line 3T3; F) Myeloid acute leukemia cell line K562; G) Myeloid acute leukemia cell line U937.

Name	Count	Percent
Amino acid transport and metabolism	599	10.18%
Carbohydrate transport and metabolism	527	8.96%
Cell cycle control, cell division, chromosome partitioning	44	0.75%
Cell motility	123	2.09%
Cell wall/membrane/envelope biogenesis	312	5.3%
Chromatin structure and dynamics	4	0.07%
Coenzyme transport and metabolism	252	4.28%
Defense mechanisms	113	1.92%
Energy production and conversion	323	5.49%
Extracellular structures	23	0.39%
Function unknown	367	6.24%
General function prediction only	654	11.11%
Inorganic ion transport and metabolism	334	5.68%
Intracellular trafficking, secretion, and vesicular transport	84	1.43%
Lipid transport and metabolism	272	4.62%
Mobilome: prophages, transposons	27	0.46%
Nucleotide transport and metabolism	116	1.97%
Posttranslational modification, protein turnover, chaperones	215	3.65%
Replication, recombination and repair	114	1.94%
Secondary metabolites biosynthesis, transport and catabolism	226	3.84%
Signal transduction mechanisms	344	5.85%
Transcription	559	9.5%
Translation, ribosomal structure and biogenesis	252	4.28%
Not in COG	1033	17.22%

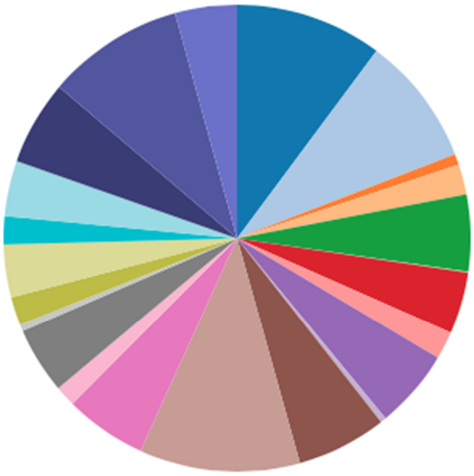

Supplementary Figure S12. Gene categories associated with general COG functional categories in *Labrenzia aggregata* USBA 371.

Supplementary Table S1. Quast statistics for assembly.

Statistic	AssemblyMEGANContigs
# contigs	53
# contigs (>= 0 bp)	65
# contigs (>= 1000 bp)	34
# contigs (>= 5000 bp)	12
# contigs (>= 10000 bp)	12
# contigs (>= 25000 bp)	11
# contigs (>= 50000 bp)	10
Largest contig	1407109
Total length	6412562
Total length (>= 0 bp)	6417675
Total length (>= 1000 bp)	6399240
Total length (>= 5000 bp)	6359191
Total length (>= 10000 bp)	6359191
Total length (>= 25000 bp)	6341785
Total length (>= 50000 bp)	6311970
N50	907758
N75	714608
L50	3
L75	5
GC (%)	59.13
Mismatches	
# N's	0
# N's per 100 kbp	0

Supplementary Table S2. Genome statistics of *Labrenzia aggregata* USBA 371

	Total Number	% of Total
**DNA, total number of bases**	6417675	100.00%
DNA coding number of bases	5667266	88.31%
DNA G+C number of bases	3795140	59.14% 1
**DNA scaffolds**	65	100.00%
**Genes total number**	5999	100.00%
Protein coding genes	5916	98.62%
Regulatory and miscellaneous features	13	0.22%4
RNA genes	70	1.17%
rRNA genes	7	0.12%
5S rRNA	2	0.03%
16S rRNA	3	0.05%
23S rRNA	2	0.03%
tRNA genes	48	0.80%
Other RNA genes	15	0.25%
Protein coding genes with function prediction	4800	80.01%
without function prediction	1116	18.60%
Protein coding genes with enzymes	1426	23.77%
Protein coding genes connected to KEGG pathways3	1760	29.34%
not connected to KEGG pathways	4156	69.28%
Protein coding genes connected to KEGG Orthology (KO)	2894	48.24%
not connected to KEGG Orthology (KO)	3022	50.38%
Protein coding genes connected to MetaCyc pathways	1239	20.65%
not connected to MetaCyc pathways	4677	77.96%
Protein coding genes with COGs3	4966	82.78%
with Pfam3	5101	85.03%
with TIGRfam3	1512	25.20%
with SMART	1290	21.50%
with SUPERFam	4843	80.73%
with CATH FunFam	4793	79.90%
in internal clusters	1775	29.59%
in Chromosomal Cassette	5885	98.10%

Chromosomal Cassettes	574	-
Biosynthetic Gene Clusters	7	-
Genes in Biosynthetic Clusters	153	2.55%
Protein coding genes coding signal peptides	599	9.98%
Protein coding genes coding transmembrane proteins	1470	24.50%
**COG clusters**	2082	41.93%
**KOG clusters**		0.00%
**Pfam clusters**	2347	46.01%
**TIGRfam clusters**	1120	74.07%

Table S3. List of proteins detected in *Labrenzia aggregata* USBA 371 using proteomic analysis

In Excel format
